# Association Between Interictal High-Frequency Oscillations and Slow Wave in Refractory Focal Epilepsy With Good Surgical Outcome

**DOI:** 10.3389/fnhum.2020.00335

**Published:** 2020-08-26

**Authors:** Guoping Ren, Jiaqing Yan, Yueqian Sun, Jiechuan Ren, Jindong Dai, Shanshan Mei, Yunlin Li, Xiaofei Wang, Xiaofeng Yang, Qun Wang

**Affiliations:** ^1^Department of Neurology, Beijing Tiantan Hospital, Capital Medical University, Beijing, China; ^2^China National Clinical Research Center for Neurological Diseases, Beijing, China; ^3^College of Electrical and Control Engineering, North China University of Technology, Beijing, China; ^4^Laboratory of Brain Disorders, Collaborative Innovation Center for Brain Disorders, Beijing Institute of Brain Disorders, Capital Medical University, Ministry of Science and Technology, Beijing, China; ^5^Department of Functional Neurosurgery, Beijing Haidian Hospital, Beijing, China; ^6^Neuroelectrophysiological Laboratory, Xuanwu Hospital, Capital Medical University, Beijing, China; ^7^Guangzhou Regenerative Medicine and Health Guangdong Laboratory, Guangzhou, China

**Keywords:** high-frequency oscillations, slow wave, refractory focal epilepsy, good surgical outcome, epileptogenic zone

## Abstract

High-frequency oscillations (HFOs) have been proposed as a promising biomarker of the epileptogenic zone (EZ). But accurate delineation of EZ based on HFOs is still challenging. Our study compared HFOs from EZ and non-EZ on the basis of their associations with interictal slow waves, aiming at exploring a new way to localize EZ. Nineteen medically intractable epilepsy patients with good surgical outcome were included. Five minute interictal intracranial electroencephalography (EEG) epochs of slow-wave sleep were randomly selected; then ripples (80–200 Hz), fast ripples (FRs; 200–500 Hz), and slow waves (0.1–4 Hz) were automatically analyzed. The EZ and non-EZ were identified by resection range during the surgeries. We found that both ripples and FRs superimposed more frequently on slow waves in EZ than in non-EZ (*P* < 0.01). Although ripples preferred to occur on the down state of slow waves in both two groups, ripples in EZ tended to be closer to the down-state peak of slow wave than in non-EZ (-174 vs. -231 ms, *P* = 0.008). As for FR, no statistical difference was found between the two groups (*P* = 0.430). Additionally, slow wave-containing ripples in EZ had a steeper slope (1.7 vs. 1.5 μV/ms, *P* < 0.001) and wider distribution ratio (32.3 vs. 30.1%, *P* < 0.001) than those in the non-EZ. But for slow wave-containing FR, only a steeper slope (1.7 vs. 1.4 μV/ms, *P* < 0.001) was observed. Our study innovatively compared the different features of association between HFOs and slow wave in EZ and non-EZ from refractory focal epilepsy with good surgical outcome, proposing a new method to localize EZ and facilitating the surgical plan.

## Introduction

High-frequency oscillations (HFOs) have been proposed as a promising biomarker of the epileptogenic zone (EZ) (Bragin et al., 1999; Jirsch et al., 2006; Thomschewski et al., 2019; Frauscher, 2020). It is categorized as ripples (80–200 Hz) and fast ripples (FRs; 200–500 Hz). The rate of HFOs is higher in the seizure onset zone or EZ than that outside these areas, and removal of tissue containing pathological HFOs is associated with good surgical outcome (Jacobs et al., 2010; Thomschewski et al., 2019). However, HFOs occur both inside and outside the EZ (Sakuraba et al., 2016; Pail et al., 2017), and FR outside the EZ may eliminate after removal of FR inside the EZ, which indicates the existence of epileptogenic network (van’t Klooster et al., 2017). Moreover, the normal brain functional activity such as learning, memory, and emotional activities can also induce physiological HFOs (Blanco et al., 2011; Buzsáki and Silva, 2012). This characteristic further improves the difficulty to accurately localize EZ on the basis of HFOs in clinical practice.

Many researches explored the methods to precisely identify EZ on the basis of interictal HFOs. They tried to find a detection threshold in order to detect channels with a frequent occurrence of interictal HFOs (Cho et al., 2014; Holler et al., 2015; Gonzalez Otarula et al., 2018; Jiang et al., 2018). Some studies also computed energy of HFOs to delineate EZ (Leung et al., 2015; Brazdil et al., 2017). Moreover, the suppressive effect of rapid eye movement sleep on interictal HFOs might also provide specific markers of epileptogenicity (Sakuraba et al., 2016). But the results of these methods were discordant, and the occurrence rate and energy of HFOs would be influenced by many factors, such as their locations in the brain and the physiological activity.

The relationship between slow wave and interictal HFOs was widely discussed in nearly a decade. Interictal coupling of HFOs and slow oscillations could predict the seizure-onset pattern in mesiotemporal lobe epilepsy (Amiri et al., 2019). A study in 123 patients showed that a stronger amplitude coupling between high-frequency activity (>150 Hz) and the phase of the slow wave (3–4 Hz) in non-resected tissues relative to that in resected tissues was independently associated with a reduced probability of good outcome (Motoi et al., 2018). Physiologic HFO activity increased during the “up state” and decreased during the “down state” of slow oscillations during deep sleep (Le Van Quyen et al., 2010), but epileptic HFOs appeared increasingly during the “down state” or the transition to it (Frauscher et al., 2015). By implementing the coupling to slow waves, the performance to classify epileptic or non-epileptic HFOs was enhanced (von Ellenrieder et al., 2016). However, in the researches, they focused on distinguishing HFOs from seizure onset zone, irritative zone, and normal brain rather than accurate identification of EZ. The precise localization of EZ is key to receive good surgical outcome and bring benefits to patients in clinical practice.

Therefore, we proceed this retrospective study to compare the relationship between HFOs and slow waves during non-rapid eye movement (NREM) sleep from EZ and non-EZ, aiming at developing a new method to identify EZ precisely.

## Materials and Methods

### Patient Population

We selected consecutive patients with pharmacoresistant epilepsy who went through continuous intracerebral electroencephalography (EEG) recordings and EZ removal surgery at the Epilepsy Centre of Beijing Haidian Hospital between January 2013 and December 2015. The inclusion criteria consisted of the following: (1) availability of at least one continuous whole night recording and (2) followed up at least 2 years and received surgical outcome of Engel I. The criteria for exclusion were as follows: (1) there were obvious artifacts in EEG and (2) patients who experienced two or more brain resection surgeries. Patients over 18 and the legal guardian/next of kin for those under 18 gave informed consent in agreement with the Research Ethics Board of Beijing Haidian Hospital. Patients were still under antiepileptic drug therapy at the time of the recording, but doses of the drugs were adjusted according to seizure frequency.

### Electrode Placement and Intracranial Electroencephalography Recording

Several kinds of electrodes were implanted in putative epileptogenic areas on the basis of previous non-invasive presurgical evaluation. A combination of cortical strips and grid electrodes (contact diameter 4 mm with a 2.5 mm exposure, spacing between contact centers 10 mm; Beijing Huakehengsheng Healthcare Co., Ltd., Beijing, China) and depth electrodes (1.2 mm diameter, eight contacts 2 mm in length, 10 mm between contacts; Beijing Huakehengsheng Healthcare Co., Ltd., Beijing, China) were implanted. Preimplantation magnetic resonance imaging (MRI) and postimplantation computer tomography (CT) scans were used to locate each contact anatomically along the electrode trajectory.

Data were recorded from the day after electrode implantation. Data for HFO analysis were acquired at 2,000 Hz with a 32- or 256-channel Nicolet recording system (Natus Medical Incorporated, San Carlos, CA, United States). The recording was performed in a monitoring unit under video surveillance and lasted for 2 days.

### Delineation of Epileptogenic Zone and Non-epileptogenic Zone

As a part of the clinical routine, the resection range was determined by neurologists and neurosurgeon according to long-term intracranial EEG monitoring. Removed channels were confirmed by comparing the fusion of presurgical MRI and CT and postsurgical MRI. In five subjects whose motor, somatosensory, or visual areas were covered with implanted grid electrodes, functional regions were defined by cortical mapping. Specifically, the functional areas included motor cortex (Patients 2, 5, 7, and 12), somatosensory cortex (Patients 2, 5, 7, and 15), and visual cortex (Patient 15). EZ was defined as the area of cortex that generates seizures, which should be removed to be seizure free. In our study, all the included patients had surgical outcome of Engel I; therefore, we considered the removed tissue was EZ and the non-resected area was non-EZ.

### Data Selection and High-Frequency Oscillation Detection

We chose a segment in slow-wave sleep (N3 stage of sleep) because NREM sleep is the state of vigilance that best identifies the EZ interictally (Klimes et al., 2019), and also in this period there is less muscle activity and more HFOs. The specific method was the same with our previous study (Ren et al., 2018). Then we randomly selected a 5 min segment during the slow-wave sleep period from each patient. All data were selected from interictal periods occurring at least 2 h from a seizure. Data with artifacts and noise, such as sharp transients with enormous amplitudes (higher than normal spikes) or irregular signals, were not selected. The data were transformed to a bipolar montage for further analysis. HFOs were automatically detected by our preliminary algorithm on the basis of maximum distributed peak points (Jiang et al., 2018; Ren et al., 2018). In the detector, the “false” HFOs caused by band-pass filtering of sharp transient events (Gibbs effect) were excluded (Jiang et al., 2018). As ripples and FRs have different generation mechanisms and electrophysiological characteristics (Jiruska et al., 2017), the algorithm was designed to analyze the ripple and FR separately.

### Slow-Wave Detection

Slow waves were detected in each intracranial channel separately by an automated detector. In bipolar intracranial channels, the polarity of slow wave depends on the local configuration of the cortex with respect to the electrode contacts. As physiologic activity in the gamma bands decreases during the down state of slow wave (Grenier et al., 2001; Mukovski et al., 2007), we computed the average gamma band (30–80 Hz) power in the positive and negative half-waves of slow wave to determine the polarity of the down state in each intracranial channel (von Ellenrieder et al., 2016). The polarity of the down state in each channel was selected as the one with lower average power in gamma band (von Ellenrieder et al., 2016). If the down state was the positive half-waves of slow wave, then signal of the channel will be turned into the opposite polarity. Thus, the down state of slow wave was the negative half-wave, which was same with scalp EEG.

**FIGURE 1:**
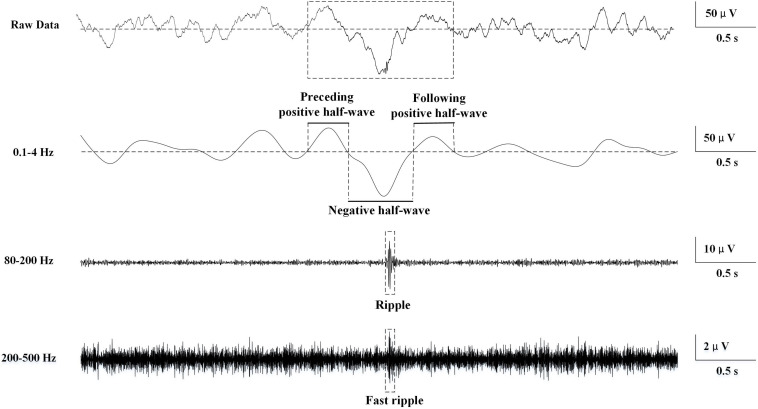
Schematic of automatic slow wave, ripple, and fast ripple (FR) detection. The first row was the raw data. The second row was band-passed from 0.1 to 4 Hz, and slow wave was automatically detected. The three phases of the slow wave were labeled. The third row was band-passed from 80 to 200 Hz, and ripple was automatically detected. The fourth row was band-passed from 200 to 500 Hz, and FR was automatically detected.

We used the zero-phase finite impulse response filter (hamming window with 881 points) to band-pass the data from 0.1 to 4 Hz. Then the slow waves were detected according to the criteria that were used previously in scalp or intracranial EEG (Valderrama et al., 2012; von Ellenrieder et al., 2016): (1) a negative wave between two succeeding zero-crossings separated by 0.125–1 s and presenting only one main peak ≤80 mV and other negative peaks not exceeding 50% of the main one (in absolute value); (2) a subsequent (or antecedent) positive wave between two succeeding zero-crossings separated by 0.125–1 s; (3) a negative-to-positive (or positive-to-negative) peak-to-peak amplitude ≥140 mV. To include only the most prominent half-waves in each channel, we only kept the 25% highest absolute amplitude. Consequently, slow waves were detected as negative half-waves, with their corresponding preceding and following positive half-waves (von Ellenrieder et al., 2016). The slow waves were divided into three parts: preceding positive half-wave, negative half-wave, and following positive half-wave (Figure 1).

### Association Between High-Frequency Oscillations and Slow Waves

Firstly, we computed the ratio of HFOs superimposed on slow waves in each channel. If HFOs occurred at the following positive half-wave of a slow wave and the preceding positive half-wave of the subsequent slow wave at the same time, they would be eliminated automatically because it was difficult to identify which slow wave did the HFOs superimpose on. Moreover, if two succeeding HFOs occur in the same part of one slow wave, only the feature of the relationship between foregoing HFO and slow wave will be computed to avoid repeatability.

Then these characteristics were explored: (1) the distribution probability of HFOs in the three phases of slow waves; (2) the timing between the onset of each HFO and the peak of the down state of the slow waves; and (3) the relationship between the relative density of HFOs and the amplitude of slow waves. The slow-wave amplitude was calculated as the percentage of the absolute value of the negative peak; and (4) the slopes and spread rates of isolated slow waves that contain HFOs. The slope was defined as the amplitude of the most negative peak divided by the time from the previous zero-crossing to the negative peak. The spread rate referred to the number ratio of channels that showed slow wave-containing HFOs simultaneously to all recording channels.

### Statistical Analysis

Data were analyzed using sigmaplot 12.0 (Systat Software, Inc., United States). The non-normal distribution data were compared by Mann–Whitney rank sum test. The composition ratio of HFO distribution in the three phases of slow wave in the two groups was compared by chi-square test. A *P* < 0.05 was considered statistically significant.

## Results

Nineteen subjects were included in the study (10 females) (see Table 1 for demographic and clinical data). The total number of channels was 757, with 420 (55.5%) in EZ and 337 (44.5%) in non-EZ.

**TABLE 1:** The clinical information of included patients.

ID	Gender	Age, years	MRI	Implantation sites	Removed sites
1	Male	16	L HS	L T, L LF, L H, L A	L AT, L partial H, L A
2	Male	33	Abnormal signal in R F	R MFG, R PreG, R PosG, R SFG, R Pos T, R P O	R Pos T, R P O
3	Female	25	Old bleeding foci in L P O	L F T, L T P	L P, L Wernicke zone
4	Female	14	Normal	L SFG, L MFG, L IFG, L M F, L T, L H	L SFG, L MFG, L IFG, L M F
5	Male	12	Abnormal signal in L F, L P cortex	L Lat F, L B F, L T, L I	L Lat F, L B F
6	Female	24	Normal	L M F, L SFG, L PreG, L P, L B F, L Lat F, L F, L M F	L M F, L SFG, L Pos T, L P
7	Male	11	Bilateral HS	R P, R O, R P O	R P, R O
8	Male	35	Normal	R F, R SFG, R B F, R T, RH, R A	R F, R SFG, R B F, R AT, R H, R A
9	Female	42	R HS, bilateral CA	R T, R F P, bilateral H, bilateral A	R AT, R H, R A
10	Male	24	Normal	L F, L T, L A, L H	L F, L AT
11	Female	39	Normal	Bilateral F, bilateral T, bilateral H, bilateral A	L AT, L partial H, L Lat OG
12	Male	21	Normal	Bilateral SFG, bilateral MFG, bilateral IFG, bilateral M F, bilateral B F, bilateral T, bilateral B T	L F, L M F
13	Female	36	OPCA	R L F, R M F, R B F, R T, R P, R H, R A	R Lat F, R M F, R B F
14	Male	16	Atrophy in L H and the whole cortex	Bilateral F, R B F, bilateral T, R I, L OFC, R H, R A	R B F, R I
15	Female	26	Bilateral CA	L F P, L middle T, L Pos T, L P O	L Pos T, L P O
16	Male	20	Encephalomalacia foci in L T P O	L F, L T, L P, L O	L P O
17	Female	8	Normal	R F, R O, R P, R T, R M F, R H	R P, R O
18	Female	27	Normal	L F, L P, L T, L H, L A	L F P, L Pos T
19	Female	13	AC in L T and L LF, abnormal signal in bilateral OHLV	R CS, R P, R P O, R T	R O

*L, left; HS, hippocampal sclerosis; T, temporal; LF, lateral fissure; H, hippocampus; A, amygdala; AT, anterior temporal; R, right; F, frontal; MFG, middle frontal gyrus; PreG, precentral gyrus; PosG, postcentral gyrus; SFG, superior frontal gyrus; Pos, posterior; P, parietal; O, occipital; IFG, inferior frontal gyrus; M, mesial; Lat, lateral; B, basis; I, insular; CA, cerebellar atrophy; OG, orbitofrontal gyrus; OPCA, olivo-ponto-cerebellar atrophy; OFC, orbit frontal cortex; AC, arachnoid cyst; OHLV, occipital horn of lateral ventricle; CS, central sulcus.*

### High-Frequency Oscillation Detection

**FIGURE 2:**
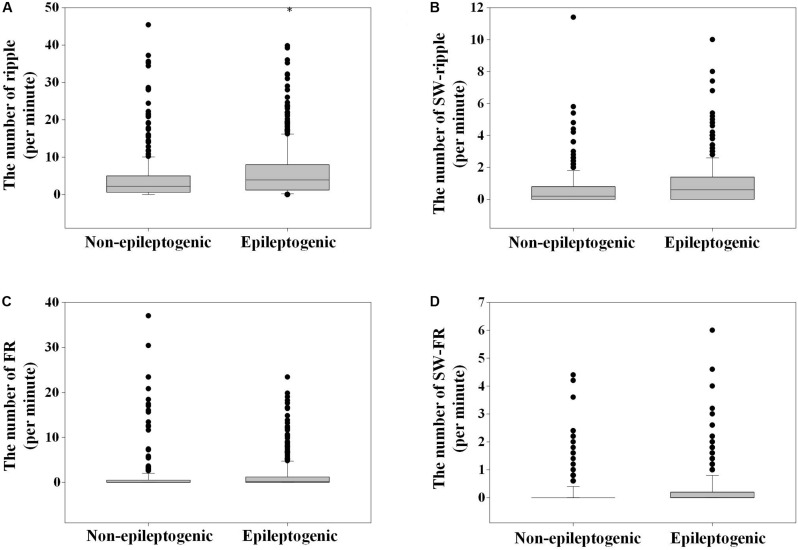
The number of ripples, fast ripple (FR), slow wave (SW)-ripple, and SW-FR in non-epileptogenic and epileptogenic zone (EZ). The rates of ripples **(A)**, SW-ripple **(B)**, FR **(C)**, and SW-FR **(D)** in EZ were significantly higher than those in non-EZ (*P* < 0.001). SW-ripple or SW-FR refers to ripple or FR that superimposed on slow wave.

A total of 20,238 ripples (12,756 in EZ and 7,482 in non-EZ) and 5,189 FRs (3,146 in EZ and 2,043 in non-EZ) were automatically detected. The median ripple rate in channels recording from the EZ was 3.9 (range 0–39.8) per minute and from the non-EZ 2.2 (range 0–45.4) per minute. In the case of FR, the median was 0.2 (range 0–23.4) per minute and 0 (range 0–37.0) per minute. The rate of both ripples and FRs in EZ were significantly higher than that in non-EZ (*P* < 0.001) (Figure 2).

### Slow-Wave Detection

A total of 20,478 slow waves (12,995 in EZ and 7,483 in non-EZ) were automatically detected. The median rate of detected slow waves in each EZ or non-EZ channel was 6.2 (range 0–18) or 4.4 (range 0–15.4) per minute, respectively. The rate of slow wave in EZ was significantly higher than that in non-EZ (*P* < 0.001).

### Association Between High-Frequency Oscillations and Slow Waves

A total of 2,303 and 1,177 ripples were found superimposed on slow wave in EZ and non-EZ, respectively. Among them, 117 (5.1%) in EZ and 74 (6.3%) in non-EZ were excluded because of overlapping on two succeeding slow waves simultaneously; 85 (3.7%) in EZ and 43 (3.7%) in non-EZ were automatically eliminated due to superimposing in the same phase of a slow wave with another ripple. Then the median slow wave-ripple (SW-ripple) rate in channels recording from the EZ was 0.6 (range 0–10) per minute and from the non-EZ 0.2 (range 0–11.4) per minute.

A total of 653 and 308 FRs were found superimposed on slow wave in EZ and non-EZ channels, respectively. Among them, 21 (3.2%) in EZ and 11 (3.6%) in non-EZ were excluded because of overlapping on two succeeding slow waves simultaneously; 56 (8.6%) in EZ and 26 (8.4%) in non-EZ were automatically eliminated due to superimposing in the same phase of a slow wave with another FR. Then the median slow wave-FR (SW-FR) rate in channels recording from the EZ was 0 (range 0–4.4) per minute and from the non-EZ 0 (range 0–6.0) per minute (Figure 2).

**FIGURE 3:**
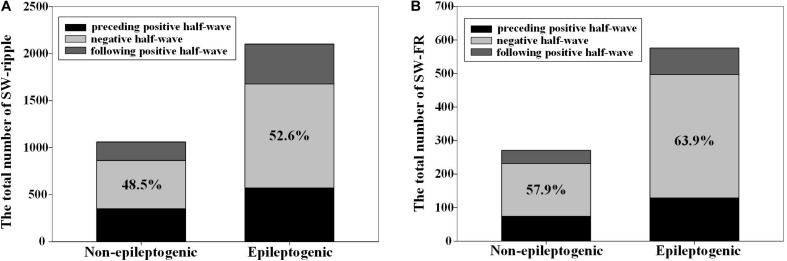
The distribution of high-frequency oscillations (HFOs) in the three different phases of slow wave. **(A)** Ripples in epileptogenic zone (EZ) and non-EZ were both likely to superimpose on the negative half-wave of the slow wave, but the composition ratio of HFO distribution in the three phases of slow wave was statistically different between EZ and non-EZ (*P* = 0.003). **(B)** Fast ripple (FR) in EZ and non-EZ tended to superimpose on the negative half-wave of the slow wave, and the composition ratio of HFO distribution in the three phases of slow wave was not statistically different between EZ and non-EZ (*P* = 0.217). Slow wave (SW)-ripple or SW-FR refers to ripple or FR that superimposed on slow wave.

The number of ripples in the three phases of slow wave were 572, 1106 (52.6%), and 423; and 350, 514 (48.5%), and 196 in EZ and non-EZ, respectively. Ripples in two groups were both likely to superimpose on the negative half-wave of the slow wave, but the composition ratio of ripple distribution in the three phases of slow wave was statistically different between the two groups (*P* = 0.003). The number of FR in the three phases of slow wave was 129, 368 (63.9%), and 79; and 74, 157 (57.9%), and 40 in EZ and non-EZ, respectively. FRs in the two groups were also likely to superimpose on the negative half-wave of the slow wave, and the composition ratio of FR distribution in the three phases of slow wave was not statistically different between the two groups (*P* = 0.217) (Figure 3).

**FIGURE 4:**
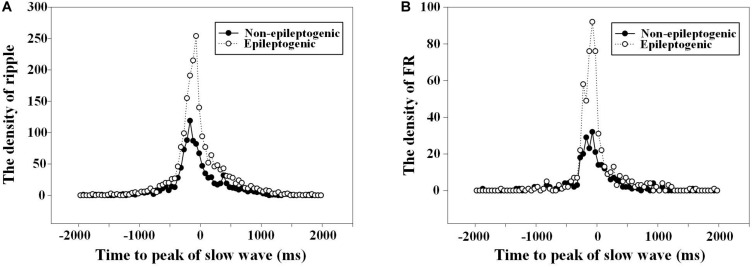
The density of high-frequency oscillations (HFOs) fluctuated with the time to the slow wave peak. **(A)** Density of ripples (80–200 Hz) per 50 ms as a function of the time to the peak (at *t* = 0) of the down state of the slow waves. The duration from the starting point of ripples to the negative peak of slow wave in epileptogenic zone (EZ) was closer than that in the non-EZ (−174.0 vs. −231.0 ms, *P* = 0.008). There was two times’ increase in the occurrence of ripples in channels recording from EZ 75 ms before the down state peak of the slow waves. **(B)** Density of fast ripple (FR) (200–500 Hz) per 50 ms as a function of the time to the peak (at *t* = 0) of the down state of the slow waves. No statistical difference was found between FR in EZ and non-EZ (−166.0 vs. −150.5 ms, *P* = 0.430).

The duration from the starting point of ripples to the negative peak of slow wave in EZ was shorter than that in the non-EZ (-174.0 vs. -231.0 ms, *P* = 0.008). As for FR, no statistical difference was found between the two groups (*P* = 0.430). Figure 4 shows the density of ripples per 50 ms as a function of the time to the peak of the down state of the slow waves.

**FIGURE 5:**
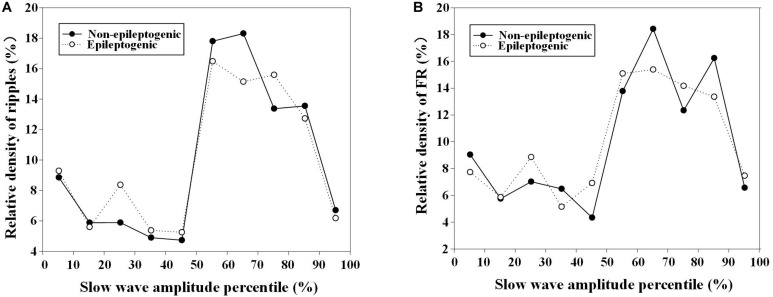
Relationship between relative density of high-frequency oscillations (HFOs) with slow-wave amplitude percentile in epileptogenic and non-epileptogenic zone (non-EZ). **(A)** The relative density of ripples at different slow-wave amplitude percentile. The relative density of ripples in EZ and non-EZ was the highest during 50–80% slow-wave amplitude percentile. **(B)** The relative density of fast ripple (FR) at different slow-wave amplitude percentile. The highest densities were distributed during 50–90%.

During the HFOs superimposed on the negative half-wave of slow waves, the relative density of ripples and FRs in EZ and non-EZ was the highest during the nearly same slow-wave amplitude percentile (50–90%) (Figure 5).

**FIGURE 6:**
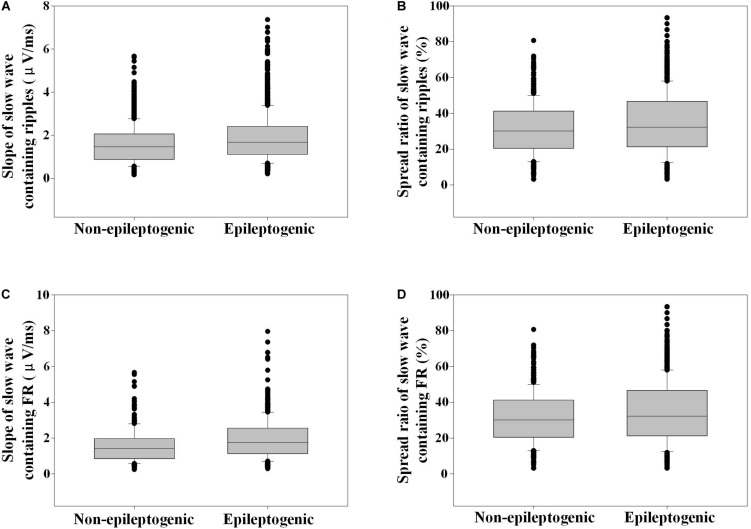
Slope and spread ratio of slow wave-containing high-frequency oscillations (HFOs) in epileptogenic and non-epileptogenic zone (non-EZ). **(A)** The slope of slow wave that contains ripples in EZ was steeper than that in the non-EZ (1.7 vs. 1.5 μV/ms, *P* < 0.001). **(B)** The spread ratio of isolated slow wave that contains ripple in EZ was higher than that in the non-EZ (32.3 vs. 30.1%, *P* < 0.001). **(C)** The slope of slow wave that contains fast ripple (FR) in EZ was steeper than that in the non-EZ (1.7 vs. 1.4 μV/ms, *P* < 0.001). **(D)** The spread ratios of isolated slow wave that contain FR were not statistically different between the two groups (31.0 vs. 28.7%, *P* = 0.788).

The slope of slow wave-containing ripples in EZ was steeper than that in the non-EZ (1.7 vs. 1.5 μV/ms, *P* < 0.001). As for FR, the slope of slow wave-containing FR in EZ was also steeper than that in the non-EZ (1.7 vs. 1.4 μV/ms, *P* < 0.001) (Figure 6).

The spread ratio of isolated slow wave that contain ripples in EZ was higher than that in the non-EZ (32.3 vs. 30.1%, *P* < 0.001). As for FR, no statistical difference was found between the two groups (*P* = 0.788) (Figure 6).

## Discussion

In this research, we compared the different associations between HFOs and the slow waves during sleep in EZ and non-EZ. We found that the rate of HFOs overlapping with slow waves was higher in EZ than that in non-EZ. HFOs tended to occur at negative half-wave of slow wave in both two zones, but ripples in EZ were much closer to the negative peak of slow wave than that in the non-EZ. Slow wave-containing ripples in EZ had a steeper slope and wider distribution ratio than those in the non-EZ. But for slow wave-containing FR, only a steeper slope was identified.

We observed that HFOs in the EZ tended to occur before the peak of the deactivated down state of the slow wave, which was concordant with the previous reports (Frauscher et al., 2015; von Ellenrieder et al., 2016; Song et al., 2017; Samiee et al., 2018). Likewise, we demonstrated the same result in the non-EZ. It is proved that some FRs in non-EZ and EZ might be related by epileptic network (van’t Klooster et al., 2017); thus, it is reasonable that they had similar characteristics when they were closed in distance. Also, the irritative zones might not always be resected completely in epilepsy surgery, so the non-EZ probably included not only normal brain regions but also irritative zones. Previous studies found that the coupling of HFOs with the phase of the slow wave in normal brain regions occurred at the transition to the “up” state (Frauscher et al., 2015; von Ellenrieder et al., 2016), but in the irritative zone, it tended to occur more often before the peak of the deactivated or down state of the slow wave (von Ellenrieder et al., 2016). Therefore, in our study, the characteristics of relationship between HFOs and slow wave in non-EZ might be the comprehensive result from irritative and normal brain zones.

Although HFOs occurred more often at negative half-wave of slow wave in both EZ and non-EZ, ripples in EZ were significantly closer to the negative peak of slow wave than that in non-EZ. The actual underlying mechanisms leading to this observation were not clear. Partial evidence suggested that slow-wave oscillations might enhance synaptic excitability and hyper-excitability, which would in turn facilitate the development of pathological hypersynchrony and eventually form epileptiform activity (Schall et al., 2008; Nazer and Dickson, 2009; Samiee et al., 2018).

Moreover, in the EZ, slow wave that contain HFOs had a steeper slope. Previously, the slope was a proxy measure of the rapidity of neural firing synchronization, which reflected synaptic strength (Esser et al., 2007; Riedner et al., 2007). Here, we calculated the slope from the preceding crossing zero to the peak of the down state. The rate at which this transition occurred across the population was determined by synaptic activity. The rate at which a population of neurons left the up state was based on the rate at which synaptic activity was removed from the network as neurons one by one entered the down state, which was in turn determined by synaptic strength (Riedner et al., 2007). Hence, it is deducible that the steeper slope of the slow wave in the EZ might indicate a higher synaptic strength, which might facilitate the occurrence of HFOs (Jiruska et al., 2017; Cepeda et al., 2020).

We also concluded that slow wave-containing ripples in EZ had wider distribution ratio than that in the non-EZ. Low-frequency rhythms, such as slow waves, were formed by a large-scale cellular network, while high-frequency rhythms were formed by a small-scale cellular network (von Stein and Sarnthein, 2000). It was demonstrated that large networks of neurons fired in synchrony and produced local-field oscillatory signals in the gamma ripple band that were triggered by slow-wave oscillations (Samiee et al., 2018). Therefore, the widespread slow waves that contain HFOs in the EZ might reflect more vulnerable tissue that could transform into epileptogenic focus.

In our study, some drawbacks existed. At primary, the number of patients was limited. Secondly, we just deduced the EZ according the resection range from patients with good surgical outcome, but the realistic size of EZ might be less than the surgery resection range. Thirdly, absolute amplitude was used to detect intracranial slow wave, which needed more discussion.

## Conclusion

In this study, we explored the association between HFOs and interictal slow wave in refractory focal epilepsy with good surgical outcome. We found that HFOs in EZ and non-EZ had different characteristics: although ripples preferred to occur on the down state of slow waves in both EZ and non-EZ, ripples in EZ tended to be closer to the down-state peak of slow wave than in non-EZ. Besides, slow wave-containing ripples in EZ had a steeper slope and wider distribution ratio than those in the non-EZ. But for slow wave-containing FR, only a steeper slope was observed. These different features between EZ and non-EZ are probably due to the synaptic hyper-excitability or the higher synaptic strength in EZ. Using the different relationship between HFOs and slow wave may be an efficient way to differ HFOs in EZ from non-EZ. More efforts are needed to verify the validity.

## Data Availability Statement

The raw data supporting the conclusions of this article will be made available by the authors, without undue reservation, to any qualified researcher.

## Ethics Statement

The studies involving human participants were reviewed and approved by the Research Ethics Board of Beijing Haidian Hospital. Written informed consent to participate in this study was provided by the participants’ legal guardian/next of kin. Written informed consent was obtained from the individual(s), and minor(s)’ legal guardian/next of kin, for the publication of any potentially identifiable images or data included in this article.

## Author Contributions

GR and JY analyzed the data. GR wrote a draft of the manuscript. All authors contributed to designing the study and collecting the data, interpreted the results, reviewed and revised the manuscript, and confirmed its final version.

## Conflict of Interest

The authors declare that the research was conducted in the absence of any commercial or financial relationships that could be construed as a potential conflict of interest.

## References

[B1] Amiri M. Frauscher B. Gotman J. (2019). Interictal coupling of HFOs and slow oscillations predicts the seizure-onset pattern in mesiotemporal lobe epilepsy. *Epilepsia* 6 1160–1170. 10.1111/epi.15541 31087662

[B2] Blanco J. A. Stead M. Krieger A. Stacey W. Maus D. Marsh E. (2011). Data mining neocortical high-frequency oscillations in epilepsy and controls. *Brain* 10(Pt. 10), 2948–2959. 10.1093/brain/awr212 21903727 PMC3187540

[B3] Bragin A. Engel J. Jr. Wilson C. L. Fried I. Buzsaki G. (1999). High-frequency oscillations in human brain. *Hippocampus* 2 137–142. 10.1002/(SICI)1098-106319999:2<137::AID-HIPO5<3.0.CO;2-0 10226774

[B4] Brazdil M. Pail M. Halamek J. Plesinger F. Cimbalnik J. Roman R. (2017). Very high-frequency oscillations: novel biomarkers of the epileptogenic zone. *Ann. Neurol.* 2 299–310. 10.1002/ana.25006 28779553

[B5] Buzsáki G. Silva F. L. (2012). High frequency oscillations in the intact brain. *Prog. Neurobiol.* 3 241–249. 10.1016/j.pneurobio.2012.02.004 22449727 PMC4895831

[B6] Cepeda C. Levinson S. Nariai H. Yazon V. W. Tran C. Barry J. (2020). Pathological high frequency oscillations associate with increased GABA synaptic activity in pediatric epilepsy surgery patients. *Neurobiol. Dis.* 134:104618. 10.1016/j.nbd.2019.104618 31629890 PMC6980668

[B7] Cho J. R. Koo D. L. Joo E. Y. Seo D. W. Hong S. C. Jiruska P. (2014). Resection of individually identified high-rate high-frequency oscillations region is associated with favorable outcome in neocortical epilepsy. *Epilepsia* 11 1872–1883. 10.1111/epi.12808 25266626

[B8] Esser S. K. Hill S. L. Tononi G. (2007). Sleep homeostasis and cortical synchronization: I. Modeling the effects of synaptic strength on sleep slow waves. *Sleep* 12 1617–1630. 10.1093/sleep/30.12.1617 18246972 PMC2276134

[B9] Frauscher B. (2020). Localizing the epileptogenic zone. *Curr. Opin. Neurol.* 2 198–206. 10.1097/WCO.0000000000000790 32049743

[B10] Frauscher B. von Ellenrieder N. Ferrari-Marinho T. Avoli M. Dubeau F. Gotman J. (2015). Facilitation of epileptic activity during sleep is mediated by high amplitude slow waves. *Brain* 138(Pt. 6), 1629–1641. 10.1093/brain/awv073 25792528 PMC4614129

[B11] Gonzalez Otarula K. A. Khoo H. M. von Ellenrieder N. Hall J. A. Dubeau F. Gotman J. (2018). Spike-related haemodynamic responses overlap with high frequency oscillations in patients with focal epilepsy. *Brain* 141 731–743. 10.1093/brain/awx383 29360943 PMC5837415

[B12] Grenier F. Timofeev I. Steriade M. (2001). Focal synchronization of ripples (80-200 Hz) in neocortex and their neuronal correlates. *J. Neurophysiol.* 4 1884–1898. 10.1152/jn.2001.86.4.1884 11600648

[B13] Holler Y. Kutil R. Klaffenbock L. Thomschewski A. Holler P. M. Bathke A. C. (2015). High-frequency oscillations in epilepsy and surgical outcome. A meta-analysis. *Front. Hum. Neurosci.* 9:574. 10.3389/fnhum.2015.00574 26539097 PMC4611152

[B14] Jacobs J. Zijlmans M. Zelmann R. Chatillon C. E. Hall J. Olivier A. (2010). High-frequency electroencephalographic oscillations correlate with outcome of epilepsy surgery. *Ann. Neurol.* 2 209–220. 10.1002/ana.21847 20225281 PMC3769290

[B15] Jiang C. Li X. Yan J. Yu T. Wang X. Ren Z. (2018). Determining the quantitative threshold of high-frequency oscillation distribution to delineate the epileptogenic zone by automated detection. *Front. Neurol.* 9:889. 10.3389/fneur.2018.00889 30483204 PMC6243027

[B16] Jirsch J. D. Urrestarazu E. LeVan P. Olivier A. Dubeau F. Gotman J. (2006). High-frequency oscillations during human focal seizures. *Brain* 129(Pt 6), 1593–1608. 10.1093/brain/awl085 16632553

[B17] Jiruska P. Alvarado-Rojas C. Schevon C. A. Staba R. Stacey W. Wendling F. (2017). Update on the mechanisms and roles of high-frequency oscillations in seizures and epileptic disorders. *Epilepsia* 8 1330–1339. 10.1111/epi.13830 28681378 PMC5554080

[B18] Klimes P. Cimbalnik J. Brazdil M. Hall J. Dubeau F. Gotman J. (2019). NREM sleep is the state of vigilance that best identifies the epileptogenic zone in the interictal electroencephalogram. *Epilepsia* 60 2404–2415. 10.1111/epi.16377 31705527

[B19] Le Van Quyen M. Staba R. Bragin A. Dickson C. Valderrama M. Fried I. (2010). Large-scale microelectrode recordings of high-frequency gamma oscillations in human cortex during sleep. *J. Neurosci.* 23 7770–7782. 10.1523/JNEUROSCI.5049-09.2010 20534826 PMC3842470

[B20] Leung H. Zhu C. X. Chan D. T. Poon W. S. Shi L. Mok V. C. (2015). Ictal high-frequency oscillations and hyperexcitability in refractory epilepsy. *Clin. Neurophysiol.* 11 2049–2057. 10.1016/j.clinph.2015.01.009 25746721

[B21] Motoi H. Miyakoshi M. Abel T. J. Jeong J. W. Nakai Y. Sugiura A. (2018). Phase-amplitude coupling between interictal high-frequency activity and slow waves in epilepsy surgery. *Epilepsia* 59 1954–1965. 10.1111/epi.14544 30146766 PMC6204289

[B22] Mukovski M. Chauvette S. Timofeev I. Volgushev M. (2007). Detection of active and silent states in neocortical neurons from the field potential signal during slow-wave sleep. *Cereb. Cortex* 2 400–414. 10.1093/cercor/bhj157 16547348

[B23] Nazer F. Dickson C. T. (2009). Slow oscillation state facilitates epileptiform events in the hippocampus. *J. Neurophysiol.* 3 1880–1889. 10.1152/jn.90795.2008 19553480

[B24] Pail M. Rehulka P. Cimbalnik J. Dolezalova I. Chrastina J. Brazdil M. (2017). Frequency-independent characteristics of high-frequency oscillations in epileptic and non-epileptic regions. *Clin. Neurophysiol.* 128 106–114. 10.1016/j.clinph.2016.10.011 27883965

[B25] Ren G. P. Yan J. Q. Yu Z. X. Wang D. Li X. N. Mei S. S. (2018). Automated detector of high frequency oscillations in epilepsy based on maximum distributed peak points. *Int. J. Neural. Syst.* 28:1750029. 10.1142/S0129065717500290 28669244

[B26] Riedner B. A. Vyazovskiy V. V. Huber R. Massimini M. Esser S. Murphy M. (2007). Sleep homeostasis and cortical synchronization: III. A high-density EEG study of sleep slow waves in humans. *Sleep* 12 1643–1657. 10.1093/sleep/30.12.1643 18246974 PMC2276133

[B27] Sakuraba R. Iwasaki M. Okumura E. Jin K. Kakisaka Y. Kato K. (2016). High frequency oscillations are less frequent but more specific to epileptogenicity during rapid eye movement sleep. *Clin. Neurophysiol.* 1 179–186. 10.1016/j.clinph.2015.05.019 26073183

[B28] Samiee S. Levesque M. Avoli M. Baillet S. (2018). Phase-amplitude coupling and epileptogenesis in an animal model of mesial temporal lobe epilepsy. *Neurobiol. Dis.* 114 111–119. 10.1016/j.nbd.2018.02.008 29486299 PMC5891384

[B29] Schall K. P. Kerber J. Dickson C. T. (2008). Rhythmic constraints on hippocampal processing: state and phase-related fluctuations of synaptic excitability during theta and the slow oscillation. *J. Neurophysiol.* 2 888–899. 10.1152/jn.00915.2007 18046004

[B30] Song I. Orosz I. Chervoneva I. Waldman Z. J. Fried I. Wu C. (2017). Bimodal coupling of ripples and slower oscillations during sleep in patients with focal epilepsy. *Epilepsia* 11 1972–1984. 10.1111/epi.13912 28948998 PMC5669821

[B31] Thomschewski A. Hincapie A. S. Frauscher B. (2019). Localization of the epileptogenic zone using high frequency oscillations. *Front. Neurol.* 10:94. 10.3389/fneur.2019.00094 30804887 PMC6378911

[B32] Valderrama M. Crepon B. Botella-Soler V. Martinerie J. Hasboun D. Alvarado-Rojas C. (2012). Human gamma oscillations during slow wave sleep. *PLoS One* 4:e33477. 10.1371/journal.pone.0033477 22496749 PMC3319559

[B33] van’t Klooster M. A. van Klink N. E. C. Zweiphenning W. Leijten F. S. S. Zelmann R. Ferrier C. H. (2017). Tailoring epilepsy surgery with fast ripples in the intraoperative electrocorticogram. *Ann. Neurol.* 5 664–676. 10.1002/ana.24928 28380659

[B34] von Ellenrieder N. Frauscher B. Dubeau F. Gotman J. (2016). Interaction with slow waves during sleep improves discrimination of physiologic and pathologic high-frequency oscillations (80-500 Hz). *Epilepsia* 6 869–878. 10.1111/epi.13380 27184021

[B35] von Stein A. Sarnthein J. (2000). Different frequencies for different scales of cortical integration: from local gamma to long range alpha/theta synchronization. *Int. J. Psychophysiol.* 3 301–313. 10.1016/s0167-8760(00)00172-0 11102669

